# Recent updates on molecular epidemiology of hepatitis C virus in Gujranwala, Pakistan

**DOI:** 10.4314/ahs.v24i4.4

**Published:** 2024-12

**Authors:** Bacha Syed Yousuf Shah, Khan Muhammad Umer, Zulfiqar Aymn, Zahid Tazeen, Ghani Muhammad Usman, Amin Iram, Shahid Muhammad, Munir Rakhtasha, Younas Saima, Vajeeha Ayesha

**Affiliations:** 1 Institute of Molecular Biology and Biotechnology, The University of Lahore, Lahore, Pakistan; 2 Centre for Applied Molecular Biology, University of the Punjab, Lahore, Pakistan; 3 Centre of Excellence in Molecular Biology (CEMB), University of the Punjab, Lahore-Pakistan

**Keywords:** Hepatitis C Virus, molecular epidemiology, genotypes

## Abstract

**Background:**

Hepatitis C infection (HCV) remains a leading cause of liver cirrhosis, posing a critical health threat worldwide.

**Objective:**

This research aimed to provide a contemporary overview of HCV infection and its genotypic distribution in Gujranwala, Punjab, Pakistan. Additionally, it sought to explore the interrelation between HCV genotypes and associated risk factors among individuals infected with HCV.

**Method:**

To examine the prevalence of antibodies against HCV, blood samples were collected from 1004 patients and tested using an immunochromatographic test (ICT). Positive ICT samples were subsequently confirmed through a chemiluminescence technique and then subjected to amplification and genotyping.

**Results:**

The study revealed that females (54.68%) were more affected by HCV than males (45.32%). Notably, the highest incidence of HCV infection (27%) was observed in the 50-59 age group. Among HCV RNA-positive patients, genotype 3a predominated, accounting for 71.81% of cases. High rate of untypable genotypes was also detected (20.82%), along with mixed (3.29%), 1a (2.09%), 1b (0.79%), 2b (0.70%), and 3b (0.50%).

**Conclusion:**

The study highlights the prevalence of genotype 3a as the most common HCV genotype in the sampled population. This information is crucial for informing public health interventions and further research in the field of HCV infection.

## Introduction

Hepatitis C infection (HCV) is one of the most common causes of progressive liver disease, with a significant impact on human health worldwide. It is the most prevalent blood-borne viral disease globally. HCV is a globally distributed human pathogen that has affected approximately 71 million people^1^, and is known as the seventh leading cause of death worldwide. Hepatocellular carcinoma and liver cirrhosis are chronic liver disorders caused by HCV^2^. HCV is a single-stranded RNA-enveloped virus with heterogeneous genetic characteristics. HCV belongs to the genus Hepacivirus, a member of the Flaviviridae family^3^. The Flaviviridae family is further categorized into three different genera: pestiviruses, flaviviruses, and hepaciviruses^4^. HCV targets normal cells, including hepatocytes and B lymphocytes^5^,^6^. The HCV virion has a diameter of about 50-60 nm and is enveloped with spherical symmetry. The surface is covered by small projection spikes and is surrounded by a prominent fringe^7^. HCV contains a small genome of about 9600 nucleotides. This genome gives rise to a polyprotein that is approximately 3010 amino acids long. Furthermore, this polypeptide is post-translationally modified by cellular and viral proteins into HCV non-structural proteins (NS2, NS3, NS4A, NS4B, NS5A, and NS5B)^8^. Due to its genetic diversity, there are 8 identified genotypes and 90 subtypes of the HCV virus^3^, ^9^.

HCV genotypes prevalence and distribution vary from region to region worldwide. There are seven HCV genotypes, each with multiple subtypes, and they are globally distributed at different rates^10^. In 2017, a global hepatitis report indicated that 71 million individuals are affected by HCV worldwide. In Egypt, the seroprevalence rate of genotype 4 is the highest among them^11^. The World Health Organization (WHO) has established a Global Health Sector Strategy (GHSS) to combat viral hepatitis by 2030. The primary goals of the GHSS for viral hepatitis are to reduce hepatitis incidence by 90% and decrease hepatitis-related mortality by up to 65% by 2030 12. Pakistan has also developd the “National Hepatitis Strategic Framework (NHSF),” which focuses on both the treatment and prevention of viral hepatitis on a national scale. In Pakistan, the main modes of hepatitis transmission are through practices such as sharing razors at barbershops, the reuse of syringes and needles, and unscreened blood transfusions during surgeries and dental procedures for different patients^13^.

Possible routes for HCV transmission include the reuse of injections among people who inject drugs (PWID), nosocomial transmission, and sexual transmission^14^. Mother-to-child transmission rates are estimated to be 5-7% and can be up to several times higher among HIV/HCV co-infected mothers. Individuals who inject drugs are considered to be at the highest risk for HCV infection^15^. Over 75% of incident infections worldwide occur among PWID. This is because HCV transmission is over ten times more likely to occur through blood-to-blood exposure than HIV transmission. In PWID, HCV infection typically precedes HIV infection^16^.

In Pakistan, there is a significant lack of correlation studies between viral load and HCV genotypes. This study also focuses on the risk factors associated with the spread of the Hepatitis C virus, as the routes of HCV transmission vary considerably among different populations. Previous studies have shown that various risk factors are linked to the transmission of HCV, including blood transfusions, road accidents, hemodialysis, dental procedures, intravenous drug misuse, beauty salons, barber shops, sexual contact, parental HCV status, tattooing, acupuncture, and abortion surgeries^17^.

In 2016, HCV infection was reported in 6% of Pakistan's population, and this percentage increased to 8.4% in 2017^18^,^19^. Among these cases, the prevalence of HCV was 51.0% among people who inject drugs (PWIDs), 1.6% in children, 10.0% in blood donors, 11.5% in the adult population, 4.65% in pregnant women, and 24.97% in patients with various diseases. Another study reported the provincial distribution of HCV prevalence as 5.46% in Punjab, 2.55% in Sindh, 25.77% in Baluchistan, 6.07% in Khyber Pakhtunkhwa, and 3.37% in FATA (formerly federally administered tribal areas), which is now under the administration of KPK 19.

Genotypes 1-3 have a worldwide distribution. A study conducted in India, a neighboring country that shares a long border with the Punjab region of Pakistan, reveals that genotype 3 is the most prevalent (63.85%), followed by genotypes 1, 4, and 6 (25.72%, 7.5%, and 2.7%) 20. The remaining genotypes show distinct geographic preferences. Genotypes 1a, 1b, 2a, and 3a are primarily distributed in high-income countries^20^, ^21^. In Japan, subtype 1b has caused 73% of infections^22^. HCV subtypes 2a and 2b are commonly found in North America and Europe, while in Northern Italy, subtype 2c is predominant^23^. Genotype 3 is most common in South-East Asia, while in Central Africa, Egypt, and the Middle East, type 4 is prevalent. Genotype 5 primarily infects the population of South Africa^24^. Genotype 6 is predominantly found in Hong Kong^25^. The existence of different HCV genotypes, such as Genotype 1, 2, 3, mixed, and untypable genotypes, reported in our study, with genotype 3a predominating, has also been observed in earlier studies conducted in Pakistan^26^. A study conducted on the population of Mardan, KPK, shows that the most predominant genotype is 3a, followed by 3b, 2a, 2b, 4a, untypable, mixed, 1a, and 1b. Another study indicated that genotype 3a is the most widely distributed, followed by 1a, mixed genotypes, 3b, and 4 genotypes^27^.

In Pakistan, the most common HCV genotype is 3a, followed by 2a, 3b, 1b, 2b, and 1a, with prevalence of 10.3%, 2.6%, 1.5%, 1.2%, and 0.5%, respectively^28^. The distribution of HCV genotypes in Pakistan includes genotype 3, genotype 1, mixed genotypes, genotype 2, untypable genotypes, genotype 4, genotype 6, and genotype 5, with prevalence rates of 78.96%, 7.03%, 5.03%, 3.81%, 3.30%, 1.59%, 0.13%, and 0.10%, respectively^29^. The most predominant HCV genotype in Lahore is genotype 3. Multiple studies conducted in Pakistan have confirmed that HCV genotype 3 is the major type, with a prevalence ranging from 75% to 90% 30, 31. Genotypes 1 and 2 are rare, with no indications of other genotypes. Within genotype 3, subtype 3a is the most prevalent (50%), followed by 3b (25%), 1b (14%), and about 10% is attributed to 1a.^32^. These results are consistent with studies conducted in other regions of Pakistan, as well as in countries bordering Pakistan, including India, Afghanistan, and Bangladesh^33^. This research aimed to provide a contemporary overview of HCV infection and its genotypic distribution in Gujranwala, Punjab, Pakistan. Additionally, it sought to explore the interrelation between HCV genotypes and associated risk factors among individuals infected with HCV subtypes.

## Materials and methods

This study was conducted at the University Institute of Medical Laboratory Technology (UIMLT), University of Lahore, from July 2019 to June 2020. The sample size consisted of 1004 samples collected from Gujranwala, the 5th largest city in Pakistan. Blood samples were collected following World Health Organization (WHO) guidelines, with the adoption of personal protective equipment and safety precautions by the Center for Disease Control (CDC) guidelines. Approximately 3-4 ml of venous blood was obtained using the venipuncture technique and placed in a 5cc syringe, then transferred to a gel tube for serum collection. Each specimen was labeled with subject name, gender, lab number, date, age, and other relevant information. The gel tube was allowed to stand for 30 minutes to clot, as it is recommended for blood to clot completely in 30-60 minutes. All infectious waste materials were appropriately disposed of in red bags, and sharp containers were sent for incineration. The inclusion criteria for this study involved HCV patients from Gujranwala, selected for genotype detection, while four samples were excluded due to hemolysis, which could introduce confounding factors into the analysis.

The initial qualitative analysis involved screening for the presence of anti-HCV antibodies using an Immunochromatographic technique (ICT) with a commercially available kit (ACON®, ACON Laboratories Inc, San Diego, CA, USA) following the manufacturer's protocol. This technique is based on chromatographic immunoassay with a test line containing recombination HCV antigen. Positive results were indicated by the appearance of a colored line on the test line, while negative results showed no color development. Positive samples were further processed for confirmation through ELISA, and molecular detection and genotyping were carried out. For the detection of HCV antibodies, Electrochemiluminescence Immuno Assay (ECLIA) was employed. This technique involves fixing the antigen to a solid surface of a well and reacting it with an enzyme-linked antibody, with the intensity of the color produced being directly proportional to the presence of antigen-antibody interaction.

RNA extraction was performed using the QIAGEN virus spin kit, involving steps like lysis, binding, washing, and elution. The viral RNA was then subjected to real-time Polymerase Chain Reaction (PCR) for amplification. The PCR program included denaturation, annealing, and extension steps with internal controls added for quality assurance. The kit used for PCR patients had a viral load sensitivity of less than 20 IU/ml. Statistical analysis was carried out using SPSS software (version 22) to analyze the recorded data. Univariate analysis was used to calculate frequencies, and the association between gender and age was observed at a 96% significant level (p<0.05).

## Results

### HCV genotype distribution of male and female

**Figure 1:**
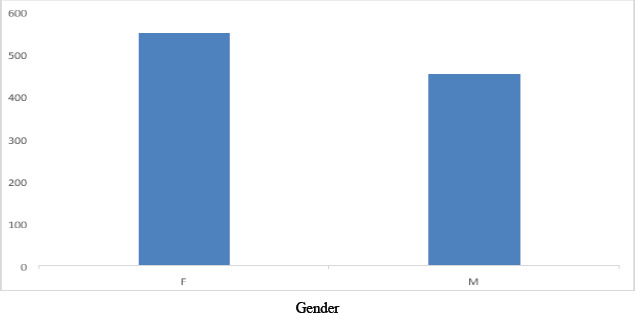
HCV genotype distribution in Male and Female

Out of the total 1004 subjects, the prevalence rate of hepatitis C virus infection was higher in females, with 549 (54.68%), compared to males, with 455 (45.32%), as shown in Figure 1. The distribution of HCV genotypes in males and females is presented in Table 1. The most prevalent subtype in Gujranwala's patients was 3a, accounting for 721 (71.81%), followed by untypable at 209 (20.82%), mixed genotype at 33 (3.29%), 1a at 21 (2.09%), 1b at 08 (0.79%), 2b at 07 (0.70%), and 3b at 0.50%. Notably, no patients with genotypes 4 and 2a were detected in this study. P-values less than 0.05 were observed for genotype 1, 2, mixed, and untypable genotype (0.004), (0.008), (0.001), and (0.000), respectively. Meanwhile, a P-value of more than 0.05 was observed for genotype 3 (0.901). The graphical representation of HCV genotype distribution is shown in Figure 1.

**Table 1:** HCV genotype distribution of male and female

Genotype/Subtypes	Male		Female		P-value	Total	
	Frequency	%	Frequency	%		Frequency	%
Genotype 1	08	1.75	21	3.82	0.004	29	2.88
1a	07	1.53	14	2.55		21	2.09
1b	01	0.21	07	1.27		08	0.79
Genotype 2	01	0.21	06	1.09	0.008	07	0.70
2a	00	00	00	00		00	00
2b	01	0.21	06	1.09		07	0.70
Genotype 3	342	75.16	384	69.94	0.901	726	72.31
3a	337	74.06	384	69.94		721	71.81
3b	05	1.09	00	00		05	0.50
Mixed genotype	14	3.07	19	3.46	0.001	33	3.29
Untypable genotype	90	19.78	119	21.67	0.000	209	20.82
Total	455	45.31	549	54.68		1004	

### HCV genotype distribution in age

Seven age groups with a 10-year difference to analyze the epidemiology and prevalence of HCV genotypes were formed, as presented in Table 2. The data reveals that the 10-19 age group had the lowest number of affected patients, with 9 (0.89%). The number of patients gradually increased, with the 20-29 age group showing 86 (8.86%) infected with HCV. In the third age group, 30-39, there were 247 (24.60%) cases, while the fourth group, 40-49, reported 250 (24.90%) cases. The fifth age group, 50-59, had the highest number of affected patients, with 271 (27.29%), which is quite alarming and raises concerns about the potential harm of increasing HCV cases. The number of patients decreased in the sixth group, 60-69, showing 104 (10.35%) patients, and in the 70-80 age group, only 17 (1.69%) patients were affected.

**Table 2:** HCV genotype distribution in different age groups

Age groups (Year)	1a	1b	2a	2b	3a	3b	4a	Mixed	Untypable	Total
10-19	00	00	00	00	07	00	00	01	01	09
20-29	05	00	00	00	70	02	00	07	22	86
30-39	02	02	00	01	189	01	00	05	47	247
40-49	03	03	00	01	180	01	00	06	56	250
50-59	07	03	00	02	194	01	00	10	54	271
60-69	04	00	00	02	73	00	00	02	23	104
70-80	00	00	00	01	08	00	00	02	06	17

### HCV genotype distribution in different region of Gujranwala

Four towns in Gujranwala, namely Khiali Shahpure, Aroop, Nandipur, and Qila Dildar Singh, were selected to determine the molecular epidemiology of HCV. Among these towns, the lowest number of affected patients was found in Khiali Shahpure town, with 234 cases (23.30%), while both Aroop and Nandipur towns reported 236 cases each (23.50%). Qila Dildar Singh town had a higher HCV prevalence, with 298 cases (29.68%). The genotypic distribution in different regions of Gujranwala is presented in Table 3. No statistical significance was observed among the different towns of Gujranwala.

**Table 3:** HCV genotype distribution in different region of Gujranwala

Genotype	Isolated from Khiali Shahpure Town	Isolated from Aroop Town	Isolated from Nandipur Town	Isolated from Qila Dildar Singh	P-value
1a	08	07	03	03	0.07
1b	03	02	01	02	0.87
2a	00	0**0**	00	00	0.00
2b	02	01	03	01	1.12
3a	172	176	168	205	0.00
3b	03	0**0**	02	00	0.37
4a	00	0**0**	00	00	0.00
Mixed	07	8	13	05	0.04
Untypable	39	42	46	82	1.98
Total	234		236	298	

### Possible routes of HCV infection

The transmissions of HCV to individuals with different genotypes are detailed in Table 4. Participants were questioned about the possible routes of infection. Out of the total, 388 (38.64%) people reported HCV infection transmission through blood transfusion, while 272 (27.09%) attributed their infection to medical and dental surgeries. Additionally, 178 (17.72%) individuals believed they were infected with HCV at beauty parlors and hair salons. A total of 102 (10.15%) respondents had no knowledge of the source of their infection, while 64 (6.37%) in the population indicated that unsafe injections were the main source of their HCV infection.

**Table 4:** Possible route for HCV infection

Genotype subtype	Blood Transfusion	Medical & Dental Surgeries	Beauty & Hair saloon	Unsafe injection	Unknown
1a	02	10	05	02	02
1b	04	02	01	01	00
2a	00	00	00	00	00
2b	04	02	01	00	00
3a	287	141	156	50	87
3b	03	02	00	00	00
4a	00	00	00	00	00
Mixed	10	13	03	02	05
No type	78	102	12	09	08
Total	388	272	178	64	102

### Viral load of HCV genotype

The study of viral load is divided into four groups: low viral loads (<10,000 IU/mL), intermediate (10,000-100,000 IU/mL), high (100,000-1,000,000 IU/mL), and extremely high (>10^^^7 IU/mL) viral loads. Low viral load was more prevalent in the population of Nandipur, with 103 cases (10.25%), followed by Aroop town with 101 cases (10.05%), Khiali Shahpur town with 93 cases (9.26%), and Qila Dildar town with 75 cases (7.47%). Overall, the ratio of individuals with low viral loads was higher in females, with 208 cases (20.71%), compared to males with 164 cases (16.33%). Intermediate viral load was the same in Aroop town and Nandipur, with 132 cases each (13.14%). In Qila Dildar Singh town, there were 125 cases (12.45%), followed by Khiali Shahpur town with 100 cases (9.96%).

High viral load was observed in Aroop town, Nandipur town, Khiali Shahpur town, and Qila Dildar Singh town, with 41 cases (4.08%), 35 cases (3.48%), 33 cases (3.28%), and 29 cases (2.88%), respectively. Extremely high viral load was the same in Khiali Shahpur and Qila Dildar town, with 2 cases (0.19%), while only 1 case (0.09%) was diagnosed in Aroop town. Extremely high viral load affected a higher number of males, with 3 cases (60%), than females, with 2 cases (40%). The detailed viral load data with genotype is presented in Table 5. The data showed statistical significance among the regions of Aroop town (0.015), Nandipur (0.000), and Qila Dildar Singh (0.001). However, the data for Khiali Shahpur was not statistically significant, with a P-value of 1.749.

**Table 5:** Viral load of HCV genotype

Genotype/Subtype	Viral Load (IU/ml)				P-value
	Low	Intermediate	High	Extremely High	
**Khiali Shahpure Town**					
Genotype 3	75	71	25	02	1.749
Other Genotype	18	29	08	00	
**Aroop Town**					
Genotype 3	68	104	28	01	0.015
Other Genotype	33	28	13	00	
**Nandipur Town**					
Genotype 3	81	88	23	00	0.000
Other Genotype	22	44	12	00	
**Qila Didar Singh Town**					
Genotype 3	54	85	20	01	0.001
Other Genotype	21	40	09	01	
Male	164	248	40	03	0.000
Female	208	241	98	2	**0**.000
Total	372	489	138	05	

## Discussion

To the best of our knowledge, this is the first comprehensive report on the molecular epidemiology of Hepatitis C virus (HCV) in Gujranwala. HCV is a blood-borne infection that is particularly common in Pakistan and is primarily spread through the sharing of brushes and needles, as well as from mother to child^34^. A total of 1004 samples from various regions of Gujranwala were collected to investigate the molecular epidemiology of Hepatitis C. Four regions were selected for this study. The study included participants of both genders, with 455 (45.31%) males and 549 (54.68%) females. Our study found that the predominant genotype in the Gujranwala population is genotype 3, with subtype 3a, which is consistent with a previous study conducted in Gujranwala^35^.

HCV genotype 3 and subtype 3a have been consistently identified as the most predominant genotypes and subtypes in various regions of Punjab. These genotypes, particularly 3 and subtype 3a, have also been reported as the predominant types in Punjab as a whole^36^. Another study conducted in Punjab also confirmd that genotype 3a is the most common^26^. According to a research conducted in Punjab, the prevalent genotype was genotype 3, with a rate of 55.10%^29^. In a 2017 study in Peshawar, it was found that the most dominant genotype in the population is 3a, accounting for 45.5% of the detected genotypes^37^. Similarly, a study conducted in Lahore revealed that genotype 3 was the most predominant, with a prevalence of 83.5%, surpassing other genotypes like 1 and 2^38^.

In our study, the most frequent and common genotype is genotype 3, followed by untypable genotypes in HCV infection. The distribution pattern of HCV genotypes in our study is nearly parallel to the findings from neighboring countries where genotype 3 is more frequent than other genotypes^39^. Notably, no patients with genotype 4a were detected in our study, whereas a study conducted in Lahore reported the presence of Genotype 4a at 12.5% and 4b at 1.2%^40^. Another study revealed that genotype 4a is predominant in Egypt and the Middle East^11^,^41^. Moreover, in our study, no patients with Genotypes 5a and 6a were isolated, while a study conducted in Peshawar recorded proportions of genotypes 5 and 6 at 0.09% and 0.22%, respectively^36^.

In this study, HCV prevalence was analyzed in a population above 10 years of age. According to our study, the most highly affected age group is 50-59 years. Another study claims that HCV infection in the Punjab population is more common among individuals aged 21-40 years^35^, Conversely, a study conducted in Peshawar reported the highest incidence in individuals aged 41-50 years (33.3%) (37). In Mardan, the number of HCV-affected individuals is higher in the middle-aged group (41-49 years)^27^.

In our study, we categorized four groups of viral load, with (10,000-100,000 IU/mL) categorized as intermediate viral load. Out of these, 489 (48.70%) people were affected by HCV with intermediate viral loads, while 372 (37.05%) people were diagnosed with low viral loads (<10,000 IU/mL). A study conducted in Punjab revealed that 29.5% of infected patients had genotype 3a with less than 600,000 IU/mL viral load, while 27.9% of patients had viral loads between 600,000-800,000 IU/mL, and 25.22% had viral loads exceeding 800,000 IU/mL(1). In Mardan, Pakistan, 10.08% of people had low viral loads, whereas 66.6% had high viral loads^27^. In Gujranwala, our study identified unsafe medical practices as the major risk factor for HCV transmission. People acquired HCV during unsafe medical procedures such as blood transfusions, unsafe injections, dental procedures, or surgeries. Visiting a hair salon, beauty parlor, and using reused blades were also significant risk factors for acquiring HCV. Another study supports these findings, emphasizing unsafe medical procedures as the main risk factor for HCV^26^, ^40^, ^41^, ^42^. The overall seroprevalence of anti-HCV in the Pakistani population among blood donors is 3.26%^43^. Regular annual physical examinations are highly recommended in Pakistan to enable early detection of HCV and facilitate timely treatment, ultimately leading to a reduction in HCV-related mortality^44^. The sample size was restricted due to the financial constraint.

## Conclusion

This study offers a comprehensive overview of the molecular epidemiology of Hepatitis C virus (HCV) in Gujranwala, Pakistan. HCV genotype 3a is the predominant subtype in this region. Furthermore, the research highlights the prevalence of genotypes, with genotype 3 being the most frequent. HCV disproportionately affects individuals in the 50-59 age group, emphasizing the need for targeted interventions within this demographic. The high prevalence of untypable HCV subtypes highlights the pressing need for the development of an enhanced genotyping system rooted in the analysis of indigenous sequence data. Moreover, the investigation identified unsafe medical practices as a major risk factor for HCV transmission in Gujranwala, underscoring the significance of improving healthcare practices and safety measures in the region.
